# Peptides Encoded by Short ORFs Control Development and Define a New Eukaryotic Gene Family

**DOI:** 10.1371/journal.pbio.0050106

**Published:** 2007-04-17

**Authors:** Máximo Ibo Galindo, José Ignacio Pueyo, Sylvaine Fouix, Sarah Anne Bishop, Juan Pablo Couso

**Affiliations:** School of Life Sciences, University of Sussex, Brighton, East Sussex, United Kingdom; Cambridge University, United Kingdom

## Abstract

Despite recent advances in developmental biology, and the sequencing and annotation of genomes, key questions regarding the organisation of cells into embryos remain. One possibility is that uncharacterised genes having nonstandard coding arrangements and functions could provide some of the answers. Here we present the characterisation of *tarsal-less (tal),* a new type of noncanonical gene that had been previously classified as a putative noncoding RNA. We show that *tal* controls gene expression and tissue folding in *Drosophila,* thus acting as a link between patterning and morphogenesis. *tal* function is mediated by several 33-nucleotide–long open reading frames (ORFs), which are translated into 11-amino-acid–long peptides. These are the shortest functional ORFs described to date, and therefore *tal* defines two novel paradigms in eukaryotic coding genes: the existence of short, unprocessed peptides with key biological functions, and their arrangement in polycistronic messengers. Our discovery of *tal*-related short ORFs in other species defines an ancient and noncanonical gene family in metazoans that represents a new class of eukaryotic genes. Our results open a new avenue for the annotation and functional analysis of genes and sequenced genomes, in which thousands of short ORFs are still uncharacterised.

## Author Summary

How cells organize into embryos remains a fundamental question in developmental biology. It is likely that significant insights into embryo development will emerge from the characterisation of novel types of genes. Yet most current genome annotation methods rely heavily on comparisons with already-known gene sequences, so genes with previously uncharacterised structures and functions can be missed. Here we present the characterisation of one of these novel genes, *tarsal-less*. *tarsal-less* has two unusual features: it contains more than one coding unit, a structure more similar to some bacterial genes; and it codes for small peptides rather than proteins. In fact, these peptides represent the smallest gene products known to date. Functional analysis of this gene in the fruit fly *Drosophila* shows that it has important functions throughout development, including tissue morphogenesis and pattern formation. We identify genes similar to *tarsal-less* in other species, and thus define a *tarsal-less*–related gene family. We expect that a combination of bioinformatic and functional methods, such as those presented in this study, will identify and characterise more genes of this type. These results suggest that hundreds of novel genes may await discovery.

## Abstract 3

A noncanonical gene*tarsal-less (tal)* is identified that links patterning and morphogenesis in*Drosophila. tal* function is mediated by short ORFs that are conserved in metazoans representing a new class of eukaryotic genes.

## Introduction

The work of the last decades has seen a breakthrough in our understanding of the genetic and molecular mechanisms of development. Classical genetic approaches have been complemented by systematic searches for new genes and their functions, resulting in an exponential increase of information. This new knowledge has filtered to related areas such as cell biology, medical research, and increasingly, evolution and population genetics. However, there still remain significant gaps in our understanding, not only of how different aspects of development such as patterning, morphogenesis, and differentiation are organised and implemented at the cellular level, but also in how these different aspects are coordinated. One exciting possibility is that new types of genes with new coding arrangements await discovery and characterisation. The number of known key regulatory genes and signalling proteins remains small, in the region of the hundreds, but sequenced and annotated genomes, including the human genome, still contain thousands of genes and transcripts without known function or sequence similarity to other genes [1–3] or are deemed RNA or noncoding genes [4].

The development of the *Drosophila* leg offers a good system in which to pursue this analysis further. Fly legs have a high density of pattern elements and a simple developmental topology, with a single main axis of patterning and growth, the PD axis [5,6]. The legs of *Drosophila* develop from presumptive organs called imaginal discs, and the morphogenesis of these discs, in particular their acquisition of a stereotyped set of folds that prefigure the morphology of the final appendage, is coordinated with patterning and growth [7,8]. An understanding of the main patterning events in leg development has recently been achieved [9,10], and a preliminary understanding of the coordination of a cell-signalling–mediated patterning event with its morphogenesis, in the development of joints, via Notch signalling, has been obtained [11–15]. More genes with well-defined morphogenetic functions await integration into this scheme [16], but the identification of further links between patterning and morphogenesis remains elusive. Our search for these links led us to the isolation and characterisation of a new *Drosophila* gene that we call *tarsal-less (tal)*. This gene expresses a 1.5-kilobase (Kb) transcript that had been classified as putatively noncoding [17,18]. It contains several open reading frames (ORFs) smaller than 50 amino acids (aa) and thus is putatively polycistronic. Our analysis shows that surprisingly, the peptides translated from ORFs of just 11 aa mediate the function of the gene. Therefore *tal* has two novel features for eukaryotic coding genes: the direct translation of short, unprocessed peptides with full biological function, and their tandem arrangement in a polycistronic messenger. We identify *tal* homologous genes in other species and observe that they define a new, noncanonical gene family of ancient origin. We expect that a combination of new bioinformatics and proteomics methods tailored to the search of peptides and small ORFs (smORFs) [19,20], plus a reassessment of classical data, will identify and characterise more new coding genes with similarly important functions in these and other areas of biology.

## Results

### Isolation and Characterisation of the *tarsal-less* Gene

**Figure 1:**
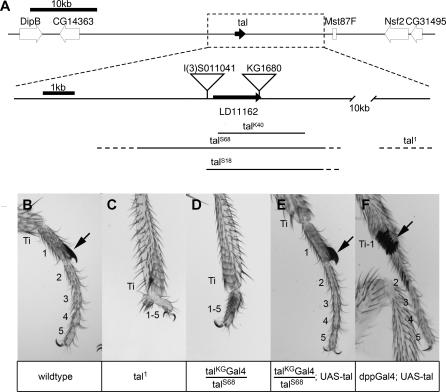
Characterisation of the *tal* Locus (A) Genomic region 87F13–15 showing the location of *tal* and neighbouring genes. The boxed area around *tal* is magnified. The inverted triangles represent the insertion sites of P elements. The solid lines indicate the fragment deleted in each mutant, with the indetermination shown as dotted lines. KG1680 and *tal^1^* are regulatory alleles for the imaginal functions, S011041 is a hypomorph, and the deletions are nulls. (B–F) Male forelegs of different genotypes. In these panels, the tibia is labelled (Ti), the tarsal segments are numbered, and the arrow points to the sex comb. (B) The tibia and five tarsal segments can be observed in the wild type. (C) In the *tal^1^* mutant, the tarsal region is vestigial and unsegmented. (D) Similar phenotype in a *tal-Gal4/tal^S68^* leg. (E) *tal-Gal4/tal^S68^; UAS-tal* shows a complete rescue of the phenotype. (F) In *dpp-Gal4; UAS-tal* ectopic expression of *tal* in the dorsal leg produces transformation of the distal tibia and fusion to tarsus 1, and ectopic sex comb in tarsi 1 and 2. These phenotypes are compatible with a transformation of tibial identity towards tarsus.

We identified the *tal* gene through a spontaneous mutant *(tal^1^)* with defective legs in which the tarsal segments [21] do not develop (Figure 1). Meiotic and deficiency mapping, followed by cytogenetic and molecular methods, revealed *tal^1^* to be a small inversion between regions 86E1,2 and 87F15. The *tal^1^* phenotype maps to the 87F15 breakpoint, to the left of the *Mst87F* gene (Figure 1A). There is no gene prediction in this region, but there is a noncoding cDNA, LD11162 [22], and two lethal P element inserts, S011041 and KG1680, located 5′ and 3′ respectively to LD11162 (Figure 1A). We found KG1680 to be allelic to *tal^1^* and to produce similar phenotypes in legs over a chromosomal deficiency for the *tal* region. These are regulatory mutants that affect only the imaginal disc function. Mobilisation of both KG1680 and S011041 insertions produced a number of alleles that all define a single complementation group. Alleles producing a deletion of the coding region for LD11162 (*tal^S68^, tal^S18^,* and *tal^K40^;* see Figure 1A) behave as nulls.

**Figure 2:**
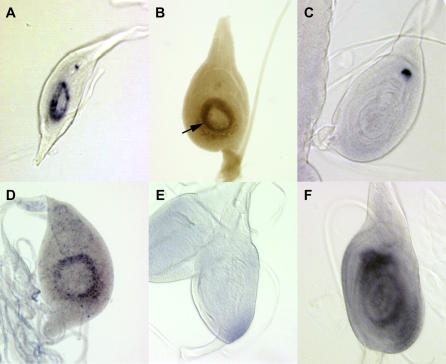
*tal* Regulates Tarsal Patterning (A) Expression of the *tal* RNA in an 84-h leg disc in a ring in the presumptive tarsal region. (B) The expression pattern of the *lacZ* gene in the reporter line l(3)S011041 faithfully reproduces the expression of the RNA. The arrow points to the tarsal fold contained in the *tal* domain. (C) By 100 h, the *tal* RNA has disappeared from the developing tarsal primordium, although it remains in a dorsal chordotonal organ. (D) *rn* RNA expression in a third instar leg disc at 90 h AEL, in the presumptive tarsal region. (E) In a *tal^1^* mutant disc, *rn* expression is abolished. (F) In a *dpp-Gal4; UAS-tal* disc at 120 h AEL, the ectopic *tal* drives expression of *rn,* at a time when neither is normally expressed.

In addition to LD11162, there are several cDNAs isolated independently [22]. We sequenced one of these, LP10384, that is identical to LD11162. In addition, a single transcript of 1.5 kb corresponding to this cDNA has been identified by Northern blots [17] and reverse-transcriptase PCR (unpublished data). The expression of this transcript is similar to the *lacZ* reporter S011041 (Figure 2A and 2B), is coincident with the regions affected in *tal* mutants (Figure 1B and 1C), and is lost in *ta1* mutants (unpublished data). To prove definitely that this transcript encodes the function of the *tal* gene, we performed a rescue experiment. The KG1680 insert was replaced by a Gal4 insert [23]. The resulting Gal4 line *(P{GaWB}tal^KG^,* subsequently referred to as *tal-Gal4)* is a regulatory viable allele similar to *tal^1^* and the KG1680 insertion, and produces a *tal* phenotype in legs (Figure 1B–1D) while simultaneously driving the expression of upstream activating sequence (UAS) constructs [24] in the *tal* pattern. We generated a construct with the full-length LP10384 cDNA downstream of a UAS promoter *(UAS-tal)* and tried to rescue mutant animals of the genotype *tal-Gal4/tal^S68^* by introducing this *UAS-tal* construct. In these *tal-Gal4/tal^S68^; UAS-tal/+* animals, the phenotypes were rescued to wild type (Figure 1E). This rescue proves that the *tal* function is encoded by LP10384, which represents the *tal* RNA. Moreover, ectopic expression of *UAS-tal* produces mutant phenotypes that are consistent with *tal* being a tarsal determinant: transformation of distal tibia and fusion to tarsi, where *tal* is normally expressed (Figure 1F).

### Functions of *tal* in Development

*tal* expression in the leg has the interesting feature of being transient (Figure 2A–2C). The time of *tal* expression (from about 80 to 96 h after egg laying [AEL]) coincides with the specification of the tarsal region by the activation of specific genes in ring patterns similar to that of *tal* [9,10]. One of the genes activated transiently at this time and required for tarsal patterning is the zinc-finger transcription factor *rotund (rn)* [25]. We observe that the expression of *rn* is lost in *tal* mutants and is extended following ectopic expression of *UAS-tal* (Figure 2D–2F). In contrast, loss or excess of function of *rn* (induced with a *UAS-rn* construct) has no effect on *tal* expression (unpublished data). These results show that the *rn* gene is a downstream target of *tal*.

**Figure 3:**
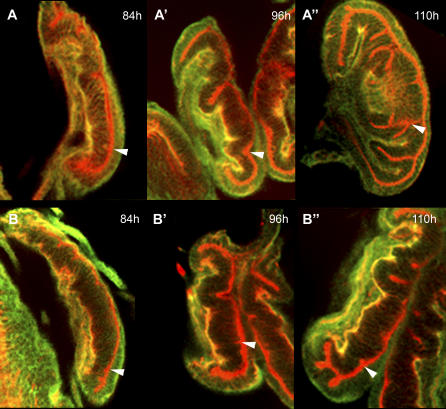
*tal* Has a Morphogenetic Function Optical sections of the third instar leg imaginal discs. The discs are shown in a side view with dorsal up and distal to the right, and the tissue morphology is revealed by phalloidin-rhodamine (red) staining of the actin cytoskeleton and anti-β-integrin (green, yellow overlap) staining of basal membranes. The position of the tarsal fold (ventral side) is indicated with an arrowhead. (A–A'') Morphological changes in a wild-type leg disc. At 84 h, the tarsal fold starts to form as an apico-basal constriction of the epithelial cells. At 96 h, this constriction is followed by invagination of the cells. At 110 h, cells that originated in the tarsal fold form secondary folds that constitute the primordia of the tarsal segments. (B–B'') In a *tal^1^* mutant, the original tarsal constriction forms as in the wild type, but the tarsal fold never forms, and basal integrin staining remains stronger than in the wild type.

Further functions of *tal* are apparent. In *tal* mutants, the whole tarsal region is missing, a stronger phenotype than that produced by *rn* mutants [25], and anti-Caspase 3 staining reveals that this is not produced by cell death (unpublished data). *tal* expression precedes and then straddles the tarsal furrow within which the tarsal segments develop (Figures 2A, 2B, and 3) [26]. In *tal* mutant discs, the tarsal fold does not form further than a superficial constriction, subsequent tarsal folds do not form, and the tarsal region does not grow (Figure 3). Reciprocally, ectopic expression of *tal* induces the appearance of ectopic folds in legs (unpublished data). These morphogenetic phenotypes are not produced by changes of *rn* expression on its own [25], and the lack of folding is not rescued by inducing expression of *rn* in *tal* mutants.

**Figure 4:**
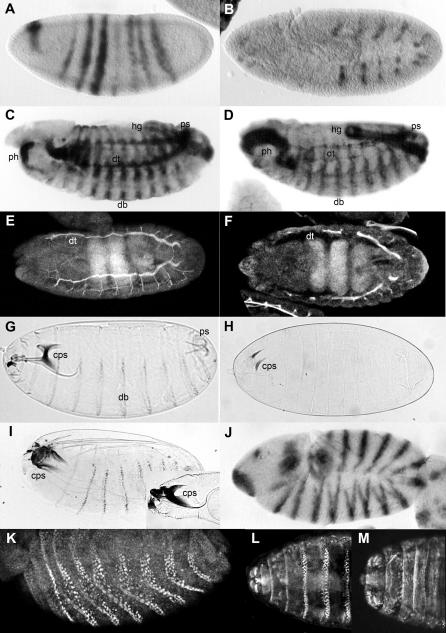
*tal* Is Required for Embryonic Development (A–D) Expression of *tal* RNA throughout embryogenesis. (A) Expression of *tal* starts in seven blastodermal stripes and a cluster of cells in the anterior part of the embryo. (B) This expression refines to the tracheal precursors by the extended germ band stage. (C and D) Later, *tal* is present in the dorsal tracheal trunks (dt), posterior spiracles (ps), pharynx (ph), hindgut (hg), and presumptive denticle belts (db). (E) Dorsal tracheal trunks (dt) in a stage 16 wild-type embryo (dorsal view) revealed by the detection of the chitin binding protein. (F) Gaps in the dorsal tracheal trunks of a *tal* mutant. (G) Wild-type embryo cuticle: cephalopharyngeal skeleton (cps), ventral denticle belts (db), and posterior spiracles (ps). (H) In *tal* null mutants, these cuticular structures are missing or reduced. (I) Ectopic *tal* expression in the head produces extra cephalopharyngeal skeleton (ventral view; inset shows lateral view). (J) Wg protein distribution in the epidermis is normal in an extended germ band *tal* mutant embryo. (K) Expression of a *shaven-baby* reporter gene in ventral epidermis is not affected in a stage 17 *tal* mutant embryo. *tal* expression is not affected either in *svb* mutants (unpublished data). (L) Ventral view of the anterior-most segments of a stage 16 wild-type embryo. The denticle cells of the epidermis accumulate tubulin bundles prior to any denticle cuticle structures being observed. (M) In *tal* mutants, these tubulin bundles do not form.

*tal* null alleles are embryonic lethal. *tal* expression in the developing embryo is initially segmental (Figure 4A; see also http://www.fruitfly.org), followed by a later and more complex pattern of expression in many organs (Figure 4B–4D). The embryonic mutant phenotypes include broken trachea, loss of cephalopharyngeal skeleton, abnormal posterior spiracles, and lack of denticle belts (Figure 4E–4H). These are the regions where *tal* is expressed from stage 13 until the end of development (Figure 4C and 4D). This phenotype is identical to a deletion of the entire 87F13–15 region, and is not enhanced by removing any putative maternal contribution in germ-line clones (unpublished data). Ectopic expression of *UAS-tal* produces reciprocal mutant phenotypes, such as extra sclerotised elements in the cephalopharyngeal skeleton (Figure 4I).

Despite the early segmental pattern of expression, *tal* mutants do not show any segmentation or homeotic phenotype (Figures 4 and S2). Therefore, the early segmental expression seems to be only a transient state to establish expression in the precursors of the tracheal system (Figure 4B). Although the mutant epidermis lacks denticle belts, segment-specific epidermal sensory organs are present, and segments are formed. Expression of markers such as *wingless* (Figure 4J), *Distal-less,* and *Ubx* (Figure S2) is normal. The late expression of *wingless* is not expanded and thus is not responsible for the observed loss of denticles [27]. Furthermore, *tal* function is independent of *shaven-baby* (Figure 4K) [28]. Altogether these results suggest that *tal* acts in parallel to the canonical denticle-patterning cascade [29]. Interestingly, *tal* mutant cells do not undergo the tubulin accumulation and cell morphology changes leading to the differentiation of denticles [30] (Figure 4L and 4M, and unpublished data).

### An 11-aa ORF Provides *tal* Function

**Figure 5:**
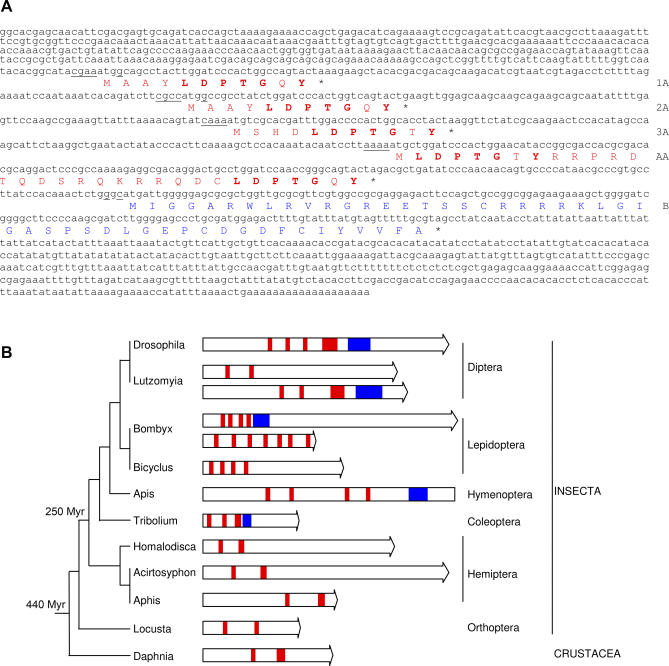
The *tal* Transcript in *Drosophila* and Other Species (A) LP10384 cDNA sequence with conceptual ORF translation; putative peptide identity is indicated on the right. Kozak consensi surrounding the start codons are underlined. Conserved domains in the type-A peptides are in bold type. (B) Graphic representation of *tal* and its homologs in other species, represented either by cDNAs (arrow ends) or genomic sequences (blunt ends). Type-A ORFs are represented by red boxes, and ORF-B by blue boxes. The *tal* gene family is at least 440 million years old and includes divergent orthologs and paralogs with different numbers of type-A ORFs. Note also that the gene duplication events in *Bombyx* and *Lutzomia* are independent. The ancestral gene had only two type-A ORFs, as shown by crustaceans and primitive insects.

Our results show that *tal* is required for several key developmental processes. The *tal* cDNA has been classified as “putatively noncoding” [17,18] on the basis of having no ORF longer than 100 aa and no known homologies. A number of candidate smORFs are present in the *tal* transcript. We will refer to these smORFs according to their sequence and position from 5′ to 3′ as 1A, 2A, 3A, AA, and B (Figure 5A). The type-A ORFs (1A, 2A, 3A, and AA) include a conserved LDPTGXY motif of 7 aa, and this motif is very strongly conserved in the cDNA of homologous genes that we have identified in other arthropods (Figure 5 and Figure S1). ORF 1A and 2A encode an identical 11-aa peptide. ORF 3A encodes another 11-aa peptide very similar to 1A. ORF AA encodes a 32-aa peptide whose N- and C-termini each contain a LDPTGXY motif (Figure 5A). ORF-B encodes a 49-aa peptide without known domains other than a poly-Arg stretch and is somehow weakly conserved in other insects (Figures 5 and S1).

**Figure 6:**
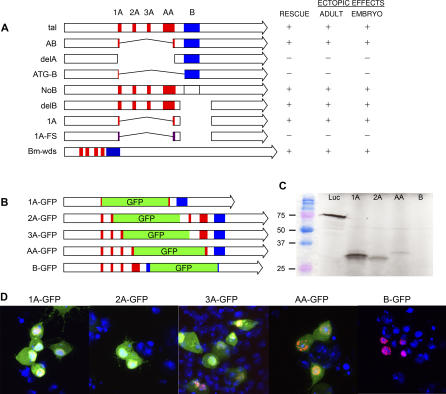
Directed Mutagenesis and Translation of *tal* (A) In these constructs, the coloured boxes indicate ORFs, and deletions are represented as empty segments. Engineered new ORFs are represented by bridged boxes. The *UAS-tal* construct contains the full cDNA and produces the complete rescue of the *tal* phenotypes and ectopic effects shown in Figures 1 and 4. Construct *AB* comprises one type-A ORF and one ORF-B, and produces the same functional effects. Construct *delA* has no type-A ORF and produces no effects. *ATG-B* forces translation of ORF-B, but still shows no effects. *NoB* has a mutation of the putative start codon of the ORF-B (empty box), thus preventing its translation, and produces the same functional effects as *UAS-tal*. *delB* has ORF-B deleted and is also fully functional. The *1A* construct, which consists of the *AB* construct plus the deletion *delB*, contains only one type-A ORF and mimics the *tal* functional effects. In the construct *1A-FS,* a single G was introduced after the start codon, causing a frameshift, which would result in the translation of a spurious 13-codon ORF (purple box). This construct is not functional. The *Bm-wds* construct contains one of the Bombyx tal full-length cDNAs and mimics the *Drosophila UAS-tal* results. (B) UAS-tal-GFP constructs tagging different ORFs, showing the in-frame insertion of the GFP coding sequence (green) (C) Peptides of expected size produced in vitro by *Luciferase* (61 kDa, control), *1A-GFP*, *2A-GFP* (28.4 kDa), and *AA-GFP* (33.1 kDa), but none by *B-GFP* (expected size, 31.6 kDa) The amount of protein produced seems to decay from 5′ to 3′ according to the ORF position, ORF 1A being the highest, and ORF AA the lowest. (D) UAS-tal-GFP constructs transfected into S2R+ cells. *1A-GFP, 2A-GFP, 3A-GFP,* and *AA-GFP* (green) are detected, but not *B-GFP*. DAPI labels nuclei (blue), and nuclear DsRed transfected cells (red).

The conservation of the aa sequences in other species suggests, but does not prove, the translation of these smORFs. With such short sequences, aa conservation cannot be distinguished easily from simple nucleotide conservation, and therefore we decided to study the functional significance of these smORFs and to obtain experimental evidence for their translation. For this, we have built upon our rescue and ectopic expression experiments that proved that *tal* is encoded by the mRNA represented by LD11162 and LP10384 (Figure 1B–1F). We have tried to rescue *tal* mutants with UAS constructs containing different directed mutations affecting specific ORFs, and in separate experiments, we have studied the ectopic effects of such constructs and compared them with those of full-length *UAS-tal*. The results are summarised in Figure 6A.

A construct containing a full-length cDNA from *Bombyx mori (Bm-wds)* produces the same effects as a full-length *Drosophila* one. This result validates the comparative results described above and also indicates that *tal* functionality lies in the ORFs, since these are the only stretches of DNA sequence conserved between *Drosophila* and *Bombyx* (Figure S1). Therefore, we next concentrated on dissecting the role of the ORF sequences in the *Drosophila* cDNA. A deletion construct *(AB)* leaving only a type-A ORF plus ORF-B is still fully functional. It can rescue *tal* mutants, and it produces the same ectopic effects as full-length *tal*. Construct *delA* deletes the type-A ORF and is just 32 base pairs (bp) shorter than *AB,* but has lost all functionality, suggesting that the type-A ORF is key for the *tal* function, and ORF-B is dispensable. It could be argued that the translation initiation context of ORF-B is too weak and that its expression requires an upstream functional type-A ORF. However, the construct *ATG-B,* in which we have put ORF-B under the control of the Tal1A initiation context, is still unable to reproduce the *tal* rescue or ectopic effects. Reciprocally, two constructs in which potential translation of ORF-B has been abolished, by either deleting it *(delB)* or by mutating its start codon *(NoB),* are fully functional, rescue *tal* mutants, and produce the same ectopic effects as full-length *UAS-tal,* including activation of *rn* expression (unpublished data). Finally, a construct containing only one type-A ORF *(1A)* is fully functional, and a one-nucleotide insertion that produces a frameshift *(1A-FS)* abolishes its functions.

Altogether, these results show that (1) an 11-aa type-A ORF provides *tal* function, and (2) ORF-B has no developmental function.

### Polycistronic Translation of *tal* RNA

These functional results indicate that *tal* function resides in the type-A ORFs, and the results with constructs *Bm-wds, 1A,* and *1A-FS* seem to exclude a model of *tal* function as a noncoding RNA. Thus we sought direct proof of *tal* translation.

The small size of the putative *tal* peptides makes them difficult to detect directly. In order to facilitate their detection in in vitro and in vivo experiments, we have tagged them by introducing the green fluorescent protein (GFP) coding sequence, minus the start and stop codons, in frame and within each of the type-A ORFs and the ORF-B (Figure 6B). Thus, the resulting fusion constructs still have the *tal* sequences relevant for translation, including the 5′ and 3′ UTRs, the initiation consensi, and start codons. Construct *1A-GFP* contains the GFP sequence within the type-A ORF of the *AB* construct, which was functional and contains the 1A translation initiation environment. *2A-GFP, 3A-GFP, AA-GFP,* and *B-GFP* contain each GFP fusion within a full-length *tal* cDNA. Expression of these constructs in a reticulocyte in vitro transcription and translation system with [^35^S]-methionine shows that the fusion peptides are expressed from the *1A-GFP, 2A-GFP,* and *AA-GFP* constructs, but not from the *B-GFP* (Figure 6C). Transfection of these constructs into *Drosophila* S2R+ cells confirmed these results and also showed translation of *3A-GFP* (Figure 6D). In all cases, we can discard the interpretation that the results are due to translation from a second methionine in the GFP sequence, not only because of the size of the fusion products obtained, but also because these putative peptides would lack the N-terminal sequences that are essential for GFP fluorescence [31].

Thus, our results show that the *tal* gene is coding, and polycistronic, because several peptides can be synthesised from a single RNA species. The type-A peptides provide the full *tal* function, and are translated both in vitro and in vivo.

## Discussion

Our results show that translation of an RNA containing smORFs of just 11 aa is required for several important processes during development. Although the *tal* cDNA contains several copies of the type-A ORFs related by a common LDPTGXY domain, a construct containing just one of them is fully functional. Small peptides are known to have important biological functions, most clearly in endocrine and neural communication [32], but in all described cases, these peptides are mature, cleaved products of a longer ORF. The originality of the *tal* gene is thus 2-fold. First, smORFs of just 33 nucleotides are fully functional and capable of translation. Second, the carefully regulated local expression of these peptides in complex patterns (as opposed to a systemic release) has important developmental functions. Our genetic and molecular analysis (Figure 1A and unpublished data) show that the *tal* genomic region contains specific regulatory sequences spread out over a minimum of 25 Kb.

### *tal* Acts during Patterning and Morphogenesis

We notice that *tal* expression and function are often associated with tissues undergoing changes of shape such as folding and invagination. The development of the fly leg is directed by a regulatory cascade involving cell signals and region-specific transcription factors [9,10,33] (reviewed in [6]). Regulatory interactions between these identity-conferring transcription factors refine and stabilise the final pattern [34,35]. This pattern is then translated into morphogenetic movements and position-specific cell differentiation programs [16,36]. *tal* seems to be an important part of the leg developmental process and to act as a link between patterning and morphogenesis. On the one hand, the transient ring of *tal* expression appears in the precise time and place to control tarsal patterning, by promoting *rn* expression and by being involved in further regulatory interactions with other leg-patterning genes (Figure 2 and unpublished data). On the other hand, *tal* controls folding of the leg tissue independently of these effects. In the wild-type leg imaginal discs, a complex morphogenetic process involving the appearance of extra folds within the tarsal furrow, in correlation with leg growth, is apparent [26]. In *tal* mutants, this morphogenetic process is compromised, whereas in excess-of-function experiments, ectopic expression of *tal* induces the appearance of ectopic folds in legs. In the mutant discs, cells undergo an apico-basal constriction, but the tarsal furrow never widens into a fold; the appearance of further tarsal sub-folds is precluded, and the presumptive tarsal region does not grow. In the embryo, *tal* expression is found in tissues of ectodermal origin that undergo an invagination without compromising their epithelial organisation, such as the foregut (and later on in its derivatives, the proventriculus and the pharynx), the hindgut, the developing trachea, and the spiracles [37]. In mutant embryos, head involution is slow, the pharynx is short and misplaced, and tracheal fusion is incomplete (Figure 4 and unpublished data). The loss of denticles in the epidermis does not seem based on alterations of the segmental patterning cascade, but on cell morphology defects that do not involve defects in apico-basal cell polarity or epidermal integrity (Figure 4 and unpublished data). Altogether, these results suggest that *tal* is required for the control of cell movements during tissue morphogenesis. Further research beyond the scope of this initial study should identify the cellular and molecular targets of this function.

### An 11-aa Peptide Defines a New Polycistronic Gene Family

Our results provide experimental evidence for function and translation of the type-A ORFs. These include the in vitro and in vivo translation assays, functional rescues, and sequence analysis. Our results therefore imply that *tal* is polycistronic, because several ORFs can be translated from a single RNA molecule. The question arises of how this can be accomplished in an eukaryotic gene, but the literature provides a possible mechanism. Polycistronic genes are known in eukaryotes including *Drosophila* [38–40], and so in principle, all *tal* ORFs could be potentially translated simultaneously. Experimental evidence supports three models for translation of polycistronic messengers in eukaryotes, namely “internal ribosomal entry sites (IRES),” “leaky scanning,” and “reinitiation” [41]. There are clear rules backed by experimental data concerning the DNA sequences and transcript structure involved in each of these models. The *tal* RNA sequence seems to exclude both the IRES and the leaky scanning possibilities. There is not enough space for IRES between the *tal* ORFs, and the initiation consensi are stronger in the 5′ ORFs than in the 3′ ones, the opposite of conditions favourable for leaky scanning. However, polycistronic translation of type-A ORFs in the *tal* transcript is possible under the reinitiation model because their spacing is between 40 and 200 bp, and the short type-A ORFs (1A to 3A) are much shorter than 35 aa. In all cases studied, the presence of 5′ ORFs has a dramatic impact on the rate of translation of the 3′ ones, leading in certain conditions, to total blockage of 3′ translation [41]. Accordingly, our in vitro translation experiment shows a diminishing amount of protein arising from each ORF, with highest levels produced by 1A, and lowest by AA (Figure 5C). We would expect, by virtue of its conserved common domain, that these translated type-A peptides will share the same functions. The presence of repeated or similar ORFs is perhaps a device to ensure enough translation of LDPTGXY-containing peptides. This hypothesis coincides with the results of our structure/function analysis, which shows that a single artificial type-A ORF suffices to provide *tal* function.

These conclusions are further corroborated by our discovery of *tal* homologous genes in other insects. These genes contain repeated copies of type-A ORFs in varying number from two (crustaceans and primitive insects) to 11 *(Bombyx mori),* and an evolutionary trend towards accumulation of more type-A ORFs, including duplications of the entire gene, is apparent. The aa sequence of these type-A ORFs is very strongly conserved in their core domain LDPTGXY. The spacing between ORFs is most compatible with the reinitiation model described above. Not only sequence, but also functionality is conserved, as indicated by the rescue of *Drosophila* mutants with a *Bombyx* cDNA. The resilience and long age of the evolutionary history of this gene family suggest, not a recently evolved curiosity of some insects, but a peptide with ancestral and current importance.

All available data suggest that the weakly conserved ORF-B is spurious or nonfunctional. In *Drosophila,* our functional analysis fails to identify any essential function for ORF-B, and both our in vitro and in vivo studies fail to detect its translation. This is in agreement with the fact that the 5′ presence of several type-A ORFs with strong initiation contexts, allied to the weakness of the context for ORF-B, does not favour the translation of ORF-B (Figure 5A). Furthermore, the size of the ORF AA is 32 aa, near the limit of 35 aa required for continued downstream reinitiation at ORF-B. In agreement with this sequence analysis, ectopic expression of the *Bombyx Bm-wds* construct containing an ORF-B in *Drosophila* does not produce any additional phenotypes when compared to those produced by the *Drosophila* constructs, indicating that the *Bombyx* ORF-B is not functional either. We would surmise that the weak conservation of ORF-B sequences is either related to some functional requirement (other than translation) for the nucleotide sequence in the region of the transcript, or pure chance.

### The *mlpt* Gene in *Tribolium*

The conservation of aa sequences has been suggested as evidence for the translation of three type-A ORFs and one ORF-B in a homologous gene called *milles-pattes (mlpt)* found in the flour beetle Tribolium castaneum [42]. These ORFs are of a similar small size as in *Drosophila,* but again such aa conservation is not conclusive evidence. In the absence of a biochemical and functional analysis of these different ORFs like the one we present here, it is difficult to guess which ORFs are translated and mediate the function of *mlpt*. The ORF-B of *mlpt* has been deemed the main functional element of the gene due to its longer length [42], but in fact, the available data belie this interpretation and favour our own conclusion of ORF-B as nonfunctional. The ORF-B of *mlpt* has no Kozak consensus at all, and its start codon overlaps with the stop codon of the previous 5′ type-A ORF, a situation that seems most unlikely to lead to ORF-B translation, even by a mechanism of readthrough as postulated [42]. Readthrough and ribosome codon slippage always proceed by skipping bases forward, rather than backwards as would be needed here. Further, ORF-B aa conservation is rather weak. Although Savard et al. [42] identify a “poly-Arg” conserved domain in alignments of selected sequences from species of only three insect orders, this conservation disappears when the comparisons are extended to further orders such as in our sequence analysis (Figures 4 and S1). We note that (1) “orphan” AUG codons are not a rare occurrence (about 500,000 in *Drosophila;* M. Ladoukakis, personal communication), and (2) that the nucleotide sequence in the ORF-B region is thymidine-poor, which produces a bias in its conceptual translation towards certain amino acids, including Arg. In addition, our analysis shows that *tal* genes without ORF-B exist, and in fact, an ORF-B is only present in some genes from holometabolous insects.

RNA interference (RNAi) analysis of the function of the whole *mlpt* transcript identifies several functions [42] that seem homologous to the one we have identified in *Drosophila,* in particular the tarsal-promoting function, and a requirement in the tracheal system. However, Savard et al. [42] also identify a “gap” and homeotic segmentation phenotypes that our expression and functional data results show to be absent in *Drosophila* (Figures 3 and S2). This functional difference might be due to the different modes of early embryonic development in *Drosophila* and *Tribolium,* which also involve a different complement of gap and maternal genes [43]. To clarify whether this segmentation function is ancestral, but has been lost in *Drosophila,* or whether it is a recently arisen specialization of *Tribolium,* will require the functional characterisation of *tal* in other insects.

### A Noncanonical Class of Eukaryotic Genes Contains smORFs

All sequenced and annotated genomes contain genes and transcripts without known function, sequence homologies, or even known protein domains. In particular, an increasing number of RNA transcripts are being classified as “noncoding” on the basis of not having ORFs longer than 50–100 aa. Furthermore, genomes contain hundreds of thousands of similarly smORFs that are systematically eliminated from gene annotations for statistical reasons. cDNA libraries and expressed sequence tag (EST) collections also discriminate against small cDNAs, perhaps losing many potential transcripts as well [44]. In the rare cases in which smORFs have been identified in longer, polycistronic messengers, studies have centred on the regulatory effect of the 5′ smORFs and resulting peptides on a standard, longer 3′ ORF. Thus, the possibility of smORFs producing peptides with important, independent functions has been largely overlooked outside of yeast, in which there is firm evidence for their existence [19]. Here we identify *tal* as a functional gene encoding only smORFs, which are translated. The *tal* type-A peptides define an ancient gene family with at least a crustacean representative (in *Daphnia*), and thus is not restricted to insects and is older than 440 million years (the estimated time for the origin of insects). We suspect that this new gene family may in fact be a representative of a new and widespread class of genes and that more genes encoding smORFs, either alone or in polycistronic messengers, await isolation and characterisation. Our analysis shows that a good cross-species sample of sequences is required to predict noncanonical peptide-coding genes, but also that these predictions must be validated by functional data, because in its absence, wrong predictions can be made. We expect that a combination of bioinformatic and functional methods tailored to the search of peptides and smORFs will identify and characterize more new gene products and eukaryotic coding genes. Preliminary results in *Drosophila* (unpublished data), yeast [19], and *Hydra* [45] suggest that hundreds of such genes may exist.

## Materials and Methods

### Fly stocks.

A synthetic deficiency for the 87F13–15 region was generated in heterozygous *Df(3)urd /Df(3)red31* flies. *dpp-Gal4* and *Dll-Gal4* were used to drive ectopic transgene expression in flies and embryos, respectively. These stocks plus l(3)S011041 ([46]) and KG1680 ([47]) are available from stock centres (http://flybase.bio.indiana.edu). The *svb^107^* enhancer trap line, which reproduces the *shaven-baby* pattern of expression [28], and the mutant allele *svb^2^* were a gift from F. Payre. Flies and embryos were mounted in Hoyer's for microscopy.

### Generation of the P{GaWB}tal^KG^ *(tal-Gal4)* line and *tal* alleles.

Replacement of the P{SuPor}KG1680 insertion by a P{GaWB} transposable element was done by mobilisation in *omb-Gal4; +/CyO Δ2–3; KG1680/TM3Sb* flies [23]. The progeny from possible replacements were screened following *UAS-GFP* expression. All replacements were precise. Mobilisation of P{lacW}l(3)S011041 and P{GaWB}*tal^KG^* was carried out with the Δ2–3 transgene. Revertants lacking white and yellow markers as appropriate were isolated. Molecular characterisation of these revertants and replacements was done by PCR, Southern blot, and sequencing as needed. *tal^S68^* and *tal^S18^* are deletions obtained by mobilisation of P{lacW}l(3)S011041, and *tal^K40^* from mobilisation of P{GaWB}*tal^KG^*.

### Immunohistochemistry and microscopy.

Developing trachea were revealed with the rhodamine-conjugated Chitin-Binding Protein (CBP at 1:500; New England Biolabs, Beverly, Massachusetts, United States). Other antibodies used were anti-β-galactosidase (1:1,000; Sigma, St. Louis, Missouri, United States; and 1:5,000; Cappel, MP Biomedicals, Solon, Ohio, United States); anti-cleaved-Caspase-3 (Asp 175: Cell Signaling Tech. at 1:250), anti-αtubulin (DM1A at 1:500; Sigma), anti-Wingless (1:50; Developmental Studies Hybridoma Bank [DSHB], Iowa City, Iowa, United States), anti-Ubx FP388 (1:20; R. White), and anti-Dll (1:2,000; I. Duncan). In developing leg discs, the actin cytoskeleton was revealed by phalloidin-rhodamine (1:40; Molecular Probes, Eugene, Oregon, United States) and basal membranes by anti-β-integrin (1:500; DSHB). Secondary antibodies conjugated to biotin, rhodamine, and FITC were used (Jackson ImmunoResearch, West Grove, Pennsylvania, United States, and Vector Laboratories, Burlingame, California, United States). Standard protocols for embryo and imaginal disc staining were followed [27]. Images were acquired and processed using a Zeiss LSM 510 confocal microscope (Carl Zeiss, Oberkochen, Germany) and LSM image software.

### In situ hybridisation.

Standard procedures were followed. DIG-labelled LP10384 was used as a *tal* RNA probe, and DIG-labelled 4H-3 *rn* cDNA fragment was used as a *rn* probe [25].

### Constructs.

The *tal* constructs are based on the LP10384 cDNA cloned in the pOT2 vector. Primer sequences and detailed strategies are available on request. The *AB* construct was made by digestion of the LP10384 cDNA with BamHI, which cuts in equivalent positions within the conserved regions of the ORF 1A and the last LDPTGXY motif of the ORF AA. The fragment containing the vector and most of the LP10384 sequence was ligated, resulting in a single type-A ORF that codes for a peptide identical to 1A. The rest of the mutant constructs were made by PCR, with primers containing directed mutations and/or restriction sites for ligation. With this strategy, we avoid any alterations to the rest of the cDNA, including UTRs and regions between the ORFs. For the *Bombyx* construct, the wdS20994 cDNA has been cloned into pPUASt. For the *1A-GFP* construct, the sequence of GFP was amplified by PCR from the pEGFP vector with internal primers so that the fragment did not contain start or stop codons, and with a BamHI adapter site. This fragment was BamHI digested and cloned into BamHI linearised AB construct. For the *2A-GFP* and *3A-GFP,* a SpeI site was introduced at the end of the LP10384 ORF 2A and ORF 3A by directed mutagenesis, then linearised, and the GFP sequence flanked by SpeI adaptors was introduced in frame. For the *AA-GFP,* a SpeI site was introduced in the middle of the ORF AA, between the two conserved LDPTGXY motifs, by directed mutagenesis, then linearised, and the GFP sequence flanked by SpeI adaptors was introduced in frame in LP10384. For the B-GFP construct, a similar strategy was employed, by introducing a KpnI site in ORF-B. For the generation of transgenic flies or transfection into S2R+ cells, these constructs were excised by double digestion with EcoRI and XhoI, and directionally cloned into pPUASt.

### In vitro transcription and translation experiments.

These were carried out using the TNT Quick Coupled Transcription/Translation reticulocyte system (Promega, Madison, Wisconsin, United States). The pool of proteins was separated by PAGE, and incorporation of [^35^S]-Met allowed the detection of the translated products by autoradiography.

### Cell culture and in vivo translation experiments.

*Drosophila* S2R+ cells were grown in Schneider's *Drosophila* medium (Invitrogen, Carlsbad, California, United States) with 10% heat-inactivated foetal bovine serum, 50-units/ml penicillin, 50-μg/ml streptomycin (Invitrogen) at 24 °C. S2R+ cells were removed from the culture flask with Trypsin-EDTA (Invitrogen). Cells were transiently transfected with 2 μg of DNA using FuGene HD (Roche, Basel, Switzerland). Plasmids transfected were p*Actin-*Gal4, pPUASt-DsRedT4NLS, and the appropriate pPUASt-tal-GFP construct. At 48 h after transfection, cells were washed in PBS, fixed for 20 min in 4% paraformaldehyde, washed twice, stained for 10 min with DAPI (Sigma), washed, and then mounted in Vectashield medium.

### DNAs and sequences.

Drosophila melanogaster cDNAs were obtained from the Berkeley *Drosophila* Genome Project (BDGP) collection [22]. *tal* cDNAs are LD11162 and LP10384. LP10384 sequencing revealed it to be identical to LD11162, with a 5′ UTR just 8 bp longer. For the phylogenetic analysis, homologous sequences were identified with the BLAST engine against several databases and obtained by different strategies. We used the following: for *Anopheles gambiae,* we obtained from the MR4 *Anopheles* repository, the cDNA 19600449643540 from the MRA-467–43 library [48]; for Lutzomyia longipalpis, two sequenced cDNAs; Bombyx mori cDNA brP0760 and EST wdS20994, which we obtained from the Silkbase EST collection [49] and sequenced; Apis mellifera genomic contig 15.24; and Tribolium castaneum gene *mlpt*. For the following species, we assembled contigs from the mentioned sequences: four Bicyclus aniana ESTs; three Homalodisca coagulata ESTs; two Aphis gossypii ESTs; three Acyrthosiphon pisum ESTs; a Locusta migratoria EST; a Daphnia pulex EST; and three genomic traces from the NCBI archive.

## Supporting Information

Figure S1 Conceptual Translation of the *tal* ORFs in Arthropod Species (22 KB DOC)

Figure S2 *tal* Is not Involved in Segmentation or Regulation of Segment Identity during Embryogenesis (4.5MB TIF)

### Accession Numbers

The National Center for Biotechnology Information (NCBI) (http://www.ncbi.nlm.nih.gov) accession numbers for the genes and gene products discussed in this paper are as follows: Acyrthosiphon pisum ESTs (CV844847, CV848262, and DY229958); Anopheles gambiae cDNA 19600449643540 (EF427621); Aphis gossypii ESTs (DR391935 and DR396643); Apis mellifera genomic contig 15.24 (NW_001253127); Bicyclus aniana ESTs (DY768921, DY768985, DY769016, and DY770310); Bombyx mori cDNA brP0760 (BP115320); Bombyx mori cDNA wdS20994 (EF427620); Daphnia pulex EST (EE682928); Daphnia pulex genomic traces from the NCBI archive (AZSH294914, AZWZ371589, and AZWZ484121); Drosophila melanogaster cDNA LD11162 (AY070879); Drosophila melanogaster cDNA LP10384 (EF427619); Homalodisca coagulata ESTs (CO641298, DN197711, and DN197836); Locusta migratoria EST (DY229958); Lutzomyia longipalpis cDNAs (AM108347 and AM108346); and *Tribolium castaneum mlpt* (AM269505).

## Abbreviations

aa: amino acid
EST: expressed sequence tag
GFP: green fluorescent protein
ORF: open reading frame
smORF: small open reading frame
UAS: upstream activating sequence
