# Supplementary material for: Peptides Encoded by Short ORFs Control Development and Define a New Eukaryotic Gene Family
Source: PLoS Biol. 2007 Apr 17;5(5):e106. doi: 10.1371/journal.pbio.0050106 (PMC1852585; doi:10.1371/journal.pbio.0050106)
Supplement: Supplementary file 1 [file pbio.0050106.sg001.doc]

# single (A) type-A ORFs

```
Drosophila melanogaster	 MAAYLDPTGQY	 MAAYLDPTGQY	    MSHDLDPTGTY
Anopheles gambiae		  MEILDPTGYY	 MAKKLDPTGHY	    MARKLDPTGHY     MAPEILDPTGYY
Lutzomyia longipalpis	(1)	 MTGILDPTEVY	 MATELDPTGHY
(2)	 MASTLDPTGHY	 MERSLDPTGMY
Bombyx mori		(1)	MDIVTLDPTGLY	 MELTLDPTGQY	    MLKVLDPTGQY       MTGLDPTEVY
	(2)	MDVKVLDPTGIY	  MLGLDPTGVY	     MYVLDPTGVY       MLVLDPTGVY
   MGLDPTGVY	  MFILDPTNVY	    MFRGLDPTGVY
Bicyclus aniana		  MFVLDPTGVY	  MFVLDPTGVY	      MDLDPTNVY      MAISLDPTGVY
Apis mellifera			 MARQLDPTGQY	 MATGLDPTGLY	     MAGLDPTGQY    MAASTGLDPTGQY
Tribolium castaneum		  MSGLDPTGLY	MDGGKLDPTGQY	MKLNGGKSLDPTGLY
Homalodisca coagulata		MFPPTLDPTGLY			  MTSDSILDPTGLY
Acyrtosiphon pisum		   MGLDPTGLY			 MCGGGPKLDPTGLY
Aphis gossypii			   MGLDPTGLY			 MCGGGPKLDPTGLY
Locusta migratoria		MGPKTLDPTGLY			  MDTPKQLDPTGLY
Daphnia pulex			 MSPKLDPTGLY	          MANTMVQQSSNVPLDPTGLY
```

AA ORF (in dipterans only)

```
Drosophila melanogaster	      MLDPTGTYRRPRDTQDSRQKRRQDC                           LDPTGQY
Anopheles gambiae		   MNRKLDPTGMYRRPGSGASSSQRSPFHHHHQQLQNHQRMPHHHQQQQQHQVKCHYLDPTGLY
Lutzomyia longipalpis		MTSTDDKLDPTGMYVRPKIEIECH                                  LDPTEYY
```

##  ORF-B

```
Drosphila melanogaster	               MIGGARWLRVRGREETSSCRRRRKLGIGASPSDLGEPCDGDFCIYVVFA
Anopheles gambiae	           MALRWWTAPQARWVRSREETSSSRRKRKFPAPGTRQQRWHRLAATVAERMNPRSGRPRMNLGVIVAYY
Lutzomyia longipalpis	 MDPCDRGAVKDLAIDTTIKAWRSVPGREETSSCRRRRKSPKMCAPTLSPATNILSSSLNK
Bombyx mori		              MCCGRRRICTGSSPAETSSCRKRRFHTDFPLC
Apis mellifera		                    MGRRVRGREETSTSNLRRTEWSIHIRKGRHRPPFTLDLKTHQ
Tribolium castaneum	                 MWHRNRGDGGRPETSSGRRRRLR
```
